# Supplementary material for: Interventions for Improving Long COVID-19 Symptomatology: A Systematic Review
Source: Viruses. 2022 Aug 24;14(9):1863. doi: 10.3390/v14091863 (PMC9502379; doi:10.3390/v14091863)
Supplement: Supplementary file 1 [file viruses-14-01863-s001.zip › viruses-1855990-supplementary.pdf]

viruses

MDPI

Supplementary Materials

Interventions for Improving Long COVID-19 Symptomatology: A Systematic Review

Supplementary Table S1. Risk of bias.

	Hawkins et al., 2022			D'Ascanio et al., 2021		
**Entry**	**Judgement**		**Support for judgement**	**Judgement**		**Support for judgement**
Random sequence generation (selection bias)	High risk of bias		Quote:This study was a randomized double blind, placebo controlled trial	High risk of bias	x	Quote:This study was a single blinded randomized-clinical trial.
	Low risk of bias	x		Low risk of bias		
	Unclear risk of bias		Comment:	Unclear risk of bias		Comment:
Allocation concealment (selection bias)	High risk of bias		Quote: Participants and participant-facing study staff were blinded to allocation of participant	High risk of bias		Quote: Not declared
	Low risk of bias	x		Low risk of bias		
	Unclear risk of bias		Comment:	Unclear risk of bias	x	Comment:
Blinding of participants and personnel (performance bias)	High risk of bias		Quote: Each participant received an unbranded bottle of the study oil, labeled with their participant ID. All study oil bottles were identical in color, shape, and size	High risk of bias		Quote: Only clinicians were blinded
	Low risk of bias	x		Low risk of bias	x	
	Unclear risk of bias		Comment:	Unclear risk of bias		Comment:
Blinding of outcome assessment (detection bias) (patient-reported outcomes)	High risk of bias		Quote:To confirm the success of blinding, participants were asked open-ended questions regarding their group assignment after the intervention concluded.	High risk of bias		Quote: Personnel was blinded regarding the outcome
	Low risk of bias	x		Low risk of bias	X	
	Unclear risk of bias		Comment:	Unclear risk of bias		Comment:
Incomplete outcome data addressed (attrition bias)	High risk of bias		Quote: Only two participants per group left the study	High risk of bias		Quote: All the participants initially included finished the study
	Low risk of bias	x		Low risk of bias	x	
	Unclear risk of bias		Comment:	Unclear risk of bias		Comment:
Selective reporting (reporting bias)	High risk of bias		Quote:	High risk of bias		Quote:
	Low risk of bias	x		Low risk of bias	x	
	Unclear risk of bias		Comment:	Unclear risk of bias		Comment:
